# Supplementary material for: The Systems Biology Research Tool: evolvable open-source software
Source: BMC Syst Biol. 2008 Jun 29;2:55. doi: 10.1186/1752-0509-2-55 (PMC2446383; doi:10.1186/1752-0509-2-55)
Supplement: Additional file 1 — SBRT Archive. An archive of the current version of the Systems Biology Research Tool. [file 1752-0509-2-55-S1.zip › sbrt-1.4.0/doc/developers_guide/api/sbrt/kernel/math/stat/comp/package-tree.html]

sbrt.kernel.math.stat.comp Class Hierarchy


|  |  |  |
| --- | --- | --- |
| **Overview** \| **Package** \| Class \| Use \| **Tree** \| **Deprecated** \| **Index** \| **Help** | |  |
| **PREV**   **NEXT** | **FRAMES**    **NO FRAMES**     **All Classes** |  |


---


## Hierarchy For Package sbrt.kernel.math.stat.comp

**Package Hierarchies:**: All Packages

---

## Class Hierarchy

- java.lang.Object
  - sbrt.kernel.math.stat.comp.**KendallTau**<V> (implements sbrt.kernel.math.stat.comp.Correlator<V,T>)
    - sbrt.kernel.math.stat.comp.**KendallTauA**<V>- sbrt.kernel.math.stat.comp.**KendallTauB**<V>
    - sbrt.kernel.math.stat.comp.**MannWhitneyResults**- sbrt.kernel.math.stat.comp.**MannWhitneyU**- sbrt.kernel.math.stat.comp.**TauResult** (implements sbrt.kernel.math.stat.comp.CorrResult)

## Interface Hierarchy

- sbrt.kernel.math.stat.comp.**Correlator**<V,T>- sbrt.kernel.math.stat.comp.**CorrResult**

---


|  |  |  |
| --- | --- | --- |
| **Overview** \| **Package** \| Class \| Use \| **Tree** \| **Deprecated** \| **Index** \| **Help** | |  |
| **PREV**   **NEXT** | **FRAMES**    **NO FRAMES**     **All Classes** |  |


---
